# EBMPracticeNet: A Bilingual National Electronic Point-Of-Care Project for Retrieval of Evidence-Based Clinical Guideline Information and Decision Support

**DOI:** 10.2196/resprot.2644

**Published:** 2013-07-10

**Authors:** Stijn Van de Velde, Robert Vander Stichele, Benjamin Fauquert, Siegfried Geens, Annemie Heselmans, Dirk Ramaekers, Ilkka Kunnamo, Bert Aertgeerts

**Affiliations:** ^1^EBMPracticeNetLeuvenBelgium; ^2^Belgian Centre for Evidence Based Medicine (CEBAM)LeuvenBelgium; ^3^CEBAM Digital Library for HealthLeuvenBelgium; ^4^Academic Center for General PracticeDepartment of Public Health and Primary CareKatholieke Universiteit LeuvenLeuvenBelgium; ^5^ZNA Hospital Network AntwerpAntwerpBelgium; ^6^DuodecimHelsinkiFinland

**Keywords:** evidence-based medicine, practice guidelines as topic, decision support systems, clinical, point-of-care systems, biomedical technology, medical informatics, information storage and retrieval, information management, ambulatory care information systems

## Abstract

**Background:**

In Belgium, the construction of a national electronic point-of-care information service, EBMPracticeNet, was initiated in 2011 to optimize quality of care by promoting evidence-based decision-making. The collaboration of the government, health care providers, evidence-based medicine (EBM) partners, and vendors of electronic health records (EHR) is unique to this project. All Belgian health care professionals get free access to an up-to-date database of validated Belgian and nearly 1000 international guidelines, incorporated in a portal that also provides EBM information from other sources than guidelines, including computerized clinical decision support that is integrated in the EHRs.

**Objective:**

The objective of this paper was to describe the development strategy, the overall content, and the management of EBMPracticeNet which may be of relevance to other health organizations creating national or regional electronic point-of-care information services.

**Methods:**

Several candidate providers of comprehensive guideline solutions were evaluated and one database was selected. Translation of the guidelines to Dutch and French was done with translation software, post-editing by translators and medical proofreading. A strategy is determined to adapt the guideline content to the Belgian context. Acceptance of the computerized clinical decision support tool has been tested and a randomized controlled trial is planned to evaluate the effect on process and patient outcomes.

**Results:**

Currently, EBMPracticeNet is in "work in progress" state. Reference is made to the results of a pilot study and to further planned research including a randomized controlled trial.

**Conclusions:**

The collaboration of government, health care providers, EBM partners, and vendors of EHRs is unique. The potential value of the project is great. The link between all the EHRs from different vendors and a national database held on a single platform that is controlled by all EBM organizations in Belgium are the strengths of EBMPracticeNet.

## Introduction

There has been an explosive growth in scientific evidence, with 75 trials and 11 systematic reviews being published a day [1]. However, the use of literature remains suboptimal because health care providers do not have the time to search actively for information or have difficulty finding the relevant evidence [2]. Too often, clinical decisions are based only upon experience, unsubstantiated routine, and opinions of experts [3,4].

Properly designed information retrieval and clinical decision support systems are now being promoted as a Global Positioning System to prevent health care providers from getting lost in clinical practice [4-6]. Such systems either use technologies, where users can pull clinical information from a database or use services that push information through reminders or alerts [7,8].

Structured clinical guidelines and computerized clinical decision support systems have the potential for improving the quality of care [9-13]. However, most of these evaluative studies focused on physician behavior or process of care rather than on the evaluation of effects on patient outcomes. Despite modern technology, it remains an important challenge to implement such systems effectively [6,14]. To be successful, it is essential that systems make clinical decision-making easier by integrating it in the clinician’s workflow the moment the clinician meets the patient. Recommendations should be generated on the fly and be action-oriented rather than mere assessments [14]. Above all, alert fatigue has to be avoided [15].

In Belgium, the construction of a national electronic point-of-care information service, EBMPracticeNet, was initiated in September 2011 to optimize quality of care by promoting evidence-based decision-making [16]. All Belgian health care professionals get free access to an up-to-date database of validated Belgian and international guidelines incorporated in a portal that also provides Evidence-based medicine (EBM) information from other sources than guidelines, including a computerized clinical decision support linked to the electronic health record (EHR). The primary focus is on general practitioners at this moment. In the second phase, there will also be a multidisciplinary focus on allied health personnel and specialist physicians. The platform is also available to patients, albeit for now not in layman’s language.

The aim of this paper is to describe the development strategy, the overall content, and the management of EBMPracticeNet.

## Methods

### Overview

**Figure 1:**
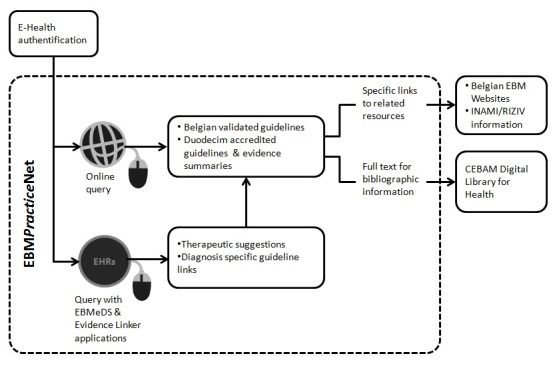
EBMPracticeNet information structure.

EBMPracticeNet was officially founded as a non-profit organization in 2011, and originated from grass roots gathering of EBM-producing organizations. This integrative cooperation project was inspired by CEBAM, the Belgian Centre for EBM and the Belgian branch of the Dutch Cochrane Collaboration. EBMPracticeNet is open to four types of organizations, namely independent producers of EBM information, disseminator organizations, user organizations, and governmental public health departments. Funding comes from the national health insurance institute (National Institute for Health and Disability Insurance, INAMI-RIZIV). The project fits within the broader range of eHealth initiatives in Belgium. The goal is to share national or adapted international EBM guidelines on one certified and secured platform (eHealth), easily accessible to different vendors of EHRs. The information structure of EBMPracticeNet is presented in Figure 1.

The guidelines database is a mix of national and international guidelines. First of all it entails about 50 Belgian guidelines, regularly updated by local Belgian Guideline producers. We supplemented this with a comprehensive database of international clinical guidelines, with the intention to adapt the content to the Belgian context. Several candidate providers of comprehensive guideline solutions were evaluated on the basis of a published review [17]. The EBM Guidelines of Duodecim Medical Publications was selected [18]. The main advantages include the strong EBM methodology, the large number of guidelines, the quality of keywords indexation, the focus on the first line level of health care, the good editorial quality, and efforts to keep the database up-to-date [17,19]. An additional argument was the formal accreditation by the UK National Health Service (NHS) of the Duodecim approach to the production of guidelines, after a formal evaluation based on the AGREE criteria [20-22]. The latter is important to demonstrate that the recommendations are trustworthy [23,24]. For the same reason, all Belgian guidelines need to be formally validated by the Belgian Centre for EBM (CEBAM), before they can be published in the database.

### Translation and Adaptation to Belgian Context

Duodecim EBM Guidelines comprise of nearly 1000 clinical guidelines and one million words. Translating this comprehensive set and adapting it to the Belgian context posed a huge challenge for EBMPracticeNet. Our first step in this process involved the translation from English to Dutch and French, two official languages of Belgium. This translation project was undertaken in cooperation with our technical partner, Iscientia IVS, a broker company for scientific information, and provider of technical platforms for scientific bibliographic information. The process was supervised by an academic institution of Applied Language Studies (Hogeschool Gent). First, the translation software, SDL Trados Studio, produced a machine translation, which was then post-edited by a human translator, a medical proofreader and a validator [25]. Machine translation was supported by a translation memory database and a terminology management system to ensure the consistent use of terms. The terminology management system used multi-term files, based on MeSH translations developed within the faculty of Applied Language Studies and based on medical glossaries available with the faculty [26]. Apart from the terminology system, the translators also consulted the InterActive Terminology for Europe multilingual term base from the European Union [27]. The post-editing of the human translators was captured in the translation memory, which increased the efficiency throughout the process. It took approximately 2000 translation hours, 500 proofreading hours and 200 validation hours per language, spread over 15 months, to accomplish the translation of the full set of guidelines. The validated versions of the translated guidelines were re-entered in the translation memory database, which will improve the quality of the translation when future updates of the international guidelines have to be translated.

Next is the adaptation of the Duodecim EBM Guidelines to the Belgian context. This process was guided by a preliminary prioritization effort. First, an inventory was made of 50 validated Belgian guidelines. The content of these Belgian guidelines was transformed in the format of the Duodecim EBM guidelines to replace the International guidelines. This involved a two-step transformation with first the production of a structured summary, and then legacy conversion into the EBM Guideline Extensible Markup Language format. Second, an additional priority list of 50 clinical topics was drafted by taking into account clinical information need (based on user surveys and epidemiological reason-for-encounter data in sentinel practice networks) and priority areas indicated by key stakeholders. The relevant guidelines for this list of priority clinical topics are now screened in close consultation with the core community of experts from the Belgian producers of EBM information [28]. The guidelines are categorized in three groups: no need for adaptation for guidelines that are trustworthy and in accordance with the Belgian context, and guidelines requiring minor or major adaptation. The guidelines requiring contextual adaptation will undergo a tailored ADAPTE procedure in collaboration with the Belgian experts [29]. Content-related remarks are followed up by Duodecim experts and editors so that the Belgian process of adaptation is synchronized with the Finnish updating cycle. We also reached out to the Belgian University Centers for Primary Care, responsible for Vocational training in General Practice, to involve a substantial group of General Practice trainees in the screening and adaptation process of additional guidelines as part of their thesis.

For the screening of the larger set of low-priority guidelines, we have invited stakeholder organizations and volunteers to screen the remaining guidelines only with regard to their compliance with the Belgian context. This process of screening and adaptation has to be completed by the end of 2015. Meanwhile, all the guidelines will be published with an explicit indication that adaptation to the Belgian context is under way. This indication will be removed when the screening (and if necessary the adaptation) is performed.

The Duodecim EBM Guidelines are revised continuously at a rate of 80 updated guidelines each trimester. This means that we need to keep up that pace of translating the updated information and screening when, and if, updated recommendations need adapting.

### Content Organization and Navigation

The user can search for information with a search engine or can browse for information using a navigation menu based on the conditions included in the database. The content and search engine are organized in such a way that they can be used during the patient encounter in a minimum of time. Implementation of direct access to the EBMPracticeNet is now a criterion in the accreditation of EHR software in Belgium.

The information in the original Duodecim EBM Guidelines database is indexed with the International Classification of Primary Care, Second edition (ICPC-2) and the International Classification of Diseases, Tenth revision (ICD-10) codes and Medical Subject Headings (MeSH) terms. The available ICD-10 and ICPC-2 codes were submitted to scrutiny and validated for the diagnostic part of the guidelines. However, additional coding is needed for process and outcome aspects. An available translation of MeSH terms in Dutch and French will be used to translate the English MeSH terms [30,31].

Based on the 1000 clinical guidelines, we will build a multilingual terminology database. Selected words and phrases will be attributed to each guideline to create an effective search engine to search the database in Dutch, French, and English.

### Portal for Other Evidence-Based Medicine Information

In addition to rapid access to practical recommendations at the point-of-care, this portal also organizes the flow of the clinical information in a chain of evidences that allows users with specific clinical questions to move efficiently from guidelines to systematic reviews and primary studies. As the time invested in these searches increase, they will typically be accessed outside the patient consultation.

- With the Duodecim EBM guidelines comes a collection of more than 4000 evidence summaries. These evidence summaries are graded statements with a short description of systematic reviews or original research [18]. In addition, the EBM guidelines information corpus includes images and videos, which are helpful in making diagnoses and carrying out procedures.
- Each guideline in the database will be linked to specific information on the websites of Belgian EBM producers. Although the Belgian EBM information is scattered across various websites, this will make it possible for the user to surf the relevant links on the site of the producers, through the EBMPracticeNet. Likewise, the guidelines will be linked to information from INAMI-RIZIV.
- Integration with the CEBAM Digital Library for Health enables the users to move from the bibliographic information provided on EBMPracticeNet to the full text of original research or systematic reviews, either in the Cochrane Library or in the large collection of scientific journals, subscribed to by the Digital Library [32,33].

### Computerized Clinical Decision Support

The computerized clinical decision support component uses the Evidence Linker technology and the Evidence-based Medicine electronic Decision Support (EBMeDS) system. The Evidence Linker is a new tool developed by the CEBAM with two General Practice trainees supervised at the KU Leuven (by BA), that provides a direct link between patient data from the EHR and guidelines for general practitioners [34].

The EBMeDS system was developed by Duodecim and its content development process is also accredited as such by the United Kingdom NHS [22,35]. The EBMeDS system receives structured patient data from EHRs and returns therapeutic suggestions and diagnosis-specific links to guidelines for a full spectrum of clinical topics.

### Promoting Implementation

To promote use of the EBMPracticeNet services, we adopt a multifaceted strategy [36]. Representatives of all local groups of family doctors receive an invitation by mail to discuss the EBMPracticeNet services at their meetings and to send in their feedback. EBMPracticeNet partners pay attention to the services in their respective publications, and organizers of EBM courses train participants in using it. Outreach visits to clinicians by the staff of an EBMPracticeNet member (ie, FARMAKA) are foreseen to give further explanations on the use of the services. Patient specific information, linked to the caregiver guidelines, will be developed in order to increase adherence to the counseling and to empower patient self-care.

### Management

The building and management of EBMPracticeNet is coordinated by a project leader, an editor-in-chief, five editors, and a secretary, all working part-time on this project and representing two full-time equivalents. The processes are being implemented in collaboration with the Belgian EBM producers, technical experts, and volunteers. Finding competent volunteers that are motivated to participate in these processes is a key factor in the sustainability of this project. The use of volunteers can include taking on the responsibility for one or several guidelines to ensure that the recommendations and their updates are in accordance with the Belgian context.

The project involves many working processes such as: information collection, processing and validation, publication and updating of the published information, and usage monitoring. An important initial effort was to describe all the key processes in Business Process Model and Notation (BPMN). The BPMN is a standard graphical notation that describes working processes in flowcharts and enables the management team to clarify and optimize processes for all stakeholders, and ensure that processes are easily transferable within the team and to the partners. We developed these flowcharts in the Open Source software Bizagi, which allows exportation to automated work flows and task lists on our editorial platform (Microsoft Sharepoint). This proved to be vital to increase the manageability of the different working processes for a small project team, collaborating with a large group of partners and volunteers.

## Results

Currently, EBMPracticeNet is still in a “work in progress” state. The use of EBMPracticeNet will be monitored in order to better meet the needs of the users and for research purposes. For this purpose, routine statistical information will be collected, such as:

- Mechanism of information retrieval: search engine, navigation system, Evidence-Linker, EBMeDS scripts.
- Type of information: search terms used, documents opened, *click-through rates* to sites of EBM producers and to CEBAM Digital Library for Health.
- User profile: type of user (health care providers, general public), language group.
- Time of use: hour and day of use, time spent in a resource.

This information is general in nature and collected anonymously, in respect of privacy regulations.

A research agenda needs to be developed to evaluate the impact of EBMPracticeNet on the care provided by Belgian health care providers. The research will comprise user-centered evaluations to analyze factors associated with failure or success; content-centered evaluation to evaluate the EBM quality; and quality and safety of care evaluation.

Preliminary results include the pilot implementation of the EBMeDS system in the EHRs of a small group of Belgian general practitioners and a quantitative and qualitative assessment of acceptance of the system has already been performed. The early adopters that responded to this survey reported a positive attitude toward this system and definitely intended to continue using it [37]. A research protocol has now been ethically approved for a randomized controlled trial (RCT) with focus on diabetes management, and is registered in clinicaltrials.gov as NCT01830569. The RCT will assess the effectiveness of the use of the EBMeDS system among Belgian general practitioners compared to the usual care process. The primary outcome measure is adherence to each of the recommendations. Secondary outcome measures include process and patient outcomes as selected from a list of quality indicators.

## Discussion

### Principal Findings and Future Directions

The prerequisite for the functioning of seamless information flows is accurate and sophisticated recording of data in the EHRs, with structured data entry, facilitated by interface terminology systems, to bridge the gap between every day medical communication and international nomenclatures and classification systems [38]. A well functioning interface terminology system should be a hybrid combination end user terminologies and one reference terminology. The end user terminology part is a unilingual lexical terminological resource (one per language), containing a selection of often used words and phrases in daily medical communication, with a splitting of polysemous meaning if present, and tagging of possible synonyms, preferably linked to National Language Processing resources such as WORDNET. The reference terminology is a multilingual resource, containing the collected concepts pertaining to a core set for medical practice, their preferred terms (for physicians and for laymen) in the different languages, a semantic bridge (word sense to concept definition), and a string bridge (word or phrase to preferred term of a concept) to the unilingual end user resources. In addition, all concepts should be mapped to several international nomenclatures (SNOMED, UMLS), classifications (ICD, ICPC), and Thesauri (MeSH). The mappings to these external systems should be the result of an expert-validated mapping effort, with qualification of the nature of the mapping (exact match; nearly exact match; imperfect but closest possible match; and impossible to match within this system) [39]. Both types of terminological resources with the interface terminology system should be represented in ISO International Standards: Lexical Markup Framework (LMF).

The LMF is used for the unilingual end user terminology and Terminological Markup Framework (TMF) is used for the multilingual reference terminology. The two resources could be managed with a Web-based Semantic Media Wiki Application, and published in Linked Open Data. Correct medical registration will optimize the functioning of automated decision support alert systems by providing both correct triggering of alerts and comments, only when necessary, and not when known exceptions are present. The coding behind correct medical registration can also provide the pathway to focused clinical questions, when practice problems surface, which halt the routine flow of the consultation process. The answer to these clinical questions can then be seamlessly provided on the guideline platform.

To facilitate information retrieval for the users, we also planned additional tools such as:

- The development of a Patient-Intervention- Comparison-Outcome interface and the complex indexing of specific practice recommendations to make the database searchable for specific patient problems [40].
- The further elaboration of the navigation menu according to taxonomy of generic clinical questions and organization of the content as a strategy to route general user questions to more specific clinical questions and focused recommendations [41].
- Specialty-specific indexing of information for several groups of allied health personnel and medical specialists.

### Conclusions

The Institute of Medicine defines Health Care Quality as the extent to which health services provided to individuals and patient populations improve desired health outcomes [42]. The care should be based on the strongest clinical evidence and provided in a technically and culturally competent manner with good communication and shared decision-making. Six aims were designed for improving the delivery of care: safety, effectiveness, patient-centeredness, efficiency, timeliness, and equitability. Improving the quality of care requires action at the micro (individual), meso (practice setting and different disciplines in primary and hospital care), and macro levels (government).

Professional behavior of caregivers consists of evidence-based practice, reflecting on their own performance, accountability, and continuous professional education. Information and communication technology (ICT) plays an important supporting role in improving the quality of care. The ICT can increase efficiency through the efficient management of resources and administrative simplification. But ICT also plays a crucial role in the effective use of treatments and patient safety (evidence-based practice) in promoting the participation of the patient and may ensure better continuous professional development and education of the caregiver.

While EBMPracticeNet is currently in “work in progress” state, the potential value of the project is great. The link between all the EHRs from different vendors with a national database held on a single platform and controlled by all EBM organizations in Belgium is the strength of EBMPracticeNet. As yet, we are not aware of an identical project in the world. The collaboration of government, health care providers, EBM partners, and vendors of EHRs is unique. With the help of national leadership in standardization and the collaboration of medical software vendors, standards can be set to facilitate the integration of different types of evidence-based and clinical information. This collaboration stems from the free delivery of independent content by the government and EBM providers, and the creativity of software vendors in creating applications for this content. A mechanism for gradual improvement of the resulting systems is the accreditation process of medical software for EHRs in which the Belgian eHealth authorities verify if the EHR fulfills the certification criteria for EHR technology.

Since Belgian EBM organizations are now formally united in EBMPracticeNet, the potential for collaboration increases. This will help reduce duplication in efforts during the development of EBM information. International collaboration on evidence synthesis and guideline development methodology, standardization of data structures, and ontologies (terminologies and their relationships) for evidence, clinical questions, recommendations and decision support, facilitated sharing of knowledge resources, and tools for staying informed about evidence will further enhance the impact of EBMPracticeNet. In addition to the EBM guidelines and EBMeDS editorial teams in Finland and Austria, the collaborative network consists of the Cochrane Collaboration, Guidelines International Network, and the GRADE Working Group.

To sustain funding for this project the impact on the quality of care will need to be demonstrated, if possible on patient outcome. The development of a research agenda is needed to verify the impact on changing clinical practice.

## Abbreviations

BPMN: Business Process Model and Notation
CEBAM: Belgian Centre for EBM
EBM: evidence-based medicine
EBMeDS: Evidence-Based Medicine electronic Decision Support
EHR: electronic health records
ICD-10: International Classification of Diseases, Tenth revision
ICPC-2: International Classification of Primary Care, Second edition
ICT: information and communication technology
INAMI-RIZIV: National Institute for Health and Disability Insurance
LMF: Lexical Markup Framework
MeSH: Medical Subject Headings
NHS: National Health Service
RCT: randomized controlled trial
TMF: Terminological Markup Framework

## References

[1] Bastian H, Glasziou P, Chalmers I (2010). Seventy-five trials and eleven systematic reviews a day: how will we ever keep up?. PLoS Med.

[2] Hannes K, Goedhuys J, Aertgeerts B (2012). Obstacles to implementing evidence-based practice in Belgium: a context-specific qualitative evidence synthesis including findings from different health care disciplines. Acta Clin Belg.

[3] Ely JW, Osheroff JA, Maviglia SM, Rosenbaum ME (2007). Patient-care questions that physicians are unable to answer. J Am Med Inform Assoc.

[4] Smith R (2010). Strategies for coping with information overload. BMJ.

[5] Moja L, Banzi R (2011). Navigators for medicine: evolution of online point-of-care evidence-based services. Int J Clin Pract.

[6] Davidoff F, Miglus J (2011). Delivering clinical evidence where it's needed: building an information system worthy of the profession. JAMA.

[7] Strayer SM, Shaughnessy AF, Yew KS, Stephens MB, Slawson DC (2010). Updating clinical knowledge: an evaluation of current information alerting services. Int J Med Inform.

[8] Moja L, Banzi R, Tagliabue L (2011). Review of "pull" point-of-care services. Int J Med Inform.

[9] Shurtz S, Foster MJ (2011). Developing and using a rubric for evaluating evidence-based medicine point-of-care tools. J Med Libr Assoc.

[10] Damiani G, Pinnarelli L, Colosimo SC, Almiento R, Sicuro L, Galasso R, Sommella L, Ricciardi W (2010). The effectiveness of computerized clinical guidelines in the process of care: a systematic review. BMC Health Serv Res.

[11] Sahota N, Lloyd R, Ramakrishna A, Mackay JA, Prorok JC, Weise-Kelly L, Navarro T, Wilczynski NL, Haynes RB, CCDSS Systematic Review Team (2011). Computerized clinical decision support systems for acute care management: a decision-maker-researcher partnership systematic review of effects on process of care and patient outcomes. Implement Sci.

[12] Heselmans A, Van de Velde S, Donceel P, Aertgeerts B, Ramaekers D (2009). Effectiveness of electronic guideline-based implementation systems in ambulatory care settings - a systematic review. Implement Sci.

[13] Lugtenberg M, Burgers JS, Westert GP (2009). Effects of evidence-based clinical practice guidelines on quality of care: a systematic review. Qual Saf Health Care.

[14] Kawamoto K, Houlihan CA, Balas EA, Lobach DF (2005). Improving clinical practice using clinical decision support systems: a systematic review of trials to identify features critical to success. BMJ.

[15] Schedlbauer A, Prasad V, Mulvaney C, Phansalkar S, Stanton W, Bates DW, Avery AJ (2009). What evidence supports the use of computerized alerts and prompts to improve clinicians' prescribing behavior?. J Am Med Inform Assoc.

[16] EBMPracticeNet.

[17] Banzi R, Liberati A, Moschetti I, Tagliabue L, Moja L (2010). A review of online evidence-based practice point-of-care information summary providers. J Med Internet Res.

[18] Varonen H, Jousimaa J, Helin-Salmivaara A, Kunnamo I (2005). Electronic primary care guidelines with links to Cochrane reviews--EBM Guidelines. Fam Pract.

[19] Banzi R, Cinquini M, Liberati A, Moschetti I, Pecoraro V, Tagliabue L, Moja L (2011). Speed of updating online evidence based point of care summaries: prospective cohort analysis. BMJ.

[20] AGREE Collaboration (2003). Development and validation of an international appraisal instrument for assessing the quality of clinical practice guidelines: the AGREE project. Qual Saf Health Care.

[21] Brouwers MC, Kho ME, Browman GP, Burgers JS, Cluzeau F, Feder G, Fervers B, Graham ID, Grimshaw J, Hanna SE, Littlejohns P, Makarski J, Zitzelsberger L, AGREE Next Steps Consortium (2010). AGREE II: advancing guideline development, reporting and evaluation in health care. J Clin Epidemiol.

[22] NICE Accreditation Decisions.

[23] Ransohoff DF, Pignone M, Sox HC (2013). How to decide whether a clinical practice guideline is trustworthy. JAMA.

[24] Alonso-Coello P, Irfan A, Solà I, Gich I, Delgado-Noguera M, Rigau D, Tort S, Bonfill X, Burgers J, Schunemann H (2010). The quality of clinical practice guidelines over the last two decades: a systematic review of guideline appraisal studies. Qual Saf Health Care.

[25] Translation Software SDL Trados Studio 2013.

[26] Terminology Centre, Faculty of applied language studies, University College Ghent 2013.

[27] InterActive Terminology for Europe 2013.

[28] Working group of Belgian producers of EBM information 2013.

[29] Fervers B, Burgers JS, Voellinger R, Brouwers M, Browman GP, Graham ID, Harrison MB, Latreille J, Mlika-Cabane N, Paquet L, Zitzelsberger L, Burnand B, ADAPTE Collaboration (2011). Guideline adaptation: an approach to enhance efficiency in guideline development and improve utilisation. BMJ Qual Saf.

[30] Zweigenbaum P (2006). ; Schulz,S.; Ruch,P, edtiors.Buysschaert J The development of a MeSH-based biomedical termbase at Hogeschool Gent.

[31] Thirion B, Pereira S, Névéol A, Dahamna B, Darmoni S (2007). French MeSH Browser: a cross-language tool to access MEDLINE/PubMed. AMIA Annu Symp Proc.

[32] CEBAM Digital Library for Health 2013.

[33] Hannes K, Vander Stichele RH, Simons E, Geens S, Goedhuys J, Aertgeerts B (2007). Implementing and optimising an Electronic Library of Health Care in Belgium: results of a pilot study. Acta Clin Belg.

[34] De Greef L, Deckers S, Lerouge F, Aertgeerts S, Geens S, Aertgeerts B (2011). Homunculus and CEBAM evidence linker. HaNu.

[35] EBMeDS Clinical Decision Support.

[36] Grol R (2001). Successes and failures in the implementation of evidence-based guidelines for clinical practice. Med Care.

[37] Heselmans A, Aertgeerts B, Donceel P, Geens S, Van de Velde S, Ramaekers D (2012). Family physicians' perceptions and use of electronic clinical decision support during the first year of implementation. J Med Syst.

[38] Rosenbloom ST, Miller RA, Johnson KB, Elkin PL, Brown SH (2008). A model for evaluating interface terminologies. J Am Med Inform Assoc.

[39] Roumier J, Vander Stichele RH, Romary L, Cardillo E (2011). Approach to the Creation of a Multilingual, Medical Interface Terminology. http://hal.inria.fr/hal-00646223_v1/.

[40] Boudin F, Nie JY, Bartlett JC, Grad R, Pluye P, Dawes M (2010). Combining classifiers for robust PICO element detection. BMC Med Inform Decis Mak.

[41] Ely JW, Osheroff JA, Gorman PN, Ebell MH, Chambliss ML, Pifer EA, Stavri PZ (2000). A taxonomy of generic clinical questions: classification study. BMJ.

[42] Corrigan JM, Donaldson MS, Kohn LT, Maguire SK, Pike KC (2001). Crossing the Quality Chasm. A New Health System for the 21st Century.

